# Antagonistic activities of cotranscriptional regulators within an early developmental window set *FLC* expression level

**DOI:** 10.1073/pnas.2102753118

**Published:** 2021-04-20

**Authors:** Michael Schon, Catherine Baxter, Congyao Xu, Balaji Enugutti, Michael D. Nodine, Caroline Dean

**Affiliations:** ^a^Gregor Mendel Institute, Vienna Biocenter, 1030 Vienna, Austria;; ^b^Department of Cell & Developmental Biology, John Innes Centre, NR4 7UH Norwich, United Kingdom;; ^c^Laboratory of Molecular Biology, Wageningen University, Wageningen, 6708 PB, The Netherlands

**Keywords:** *Arabidopsis FLC*, cotranscriptional regulation, embryogenesis, transcript isoforms, sector analysis

## Abstract

Quantitative variation in expression of the *Arabidopsis* floral repressor *FLC* influences whether plants overwinter before flowering, or have a rapid cycling habit enabling multiple generations a year. Genetic analysis has identified activators and repressors of *FLC* expression but how they interact to set expression level is poorly understood. Here, we show that antagonistic functions of the *FLC* activator FRIGIDA (FRI) and the repressor FCA, at a specific stage of embryo development, determine *FLC* expression and flowering. FRI antagonizes an FCA-induced proximal polyadenylation to increase *FLC* expression and delay flowering. Sector analysis shows that FRI activity during the early heart stage of embryo development maximally delays flowering. Opposing functions of cotranscriptional regulators during an early embryonic developmental window thus set *FLC* expression levels and determine flowering time.

Quantitative regulation of *FLC* expression influences overwintering requirement and vernalization of many Brassicaceae species. Regulators of *FLC* expression have been identified by genetic analysis in *Arabidopsis*. A series of repressors grouped in the autonomous pathway include RNA binding proteins, 3′-end processing factors, and chromatin modifiers. One of the RNA binding proteins, FCA, promotes proximal polyadenylation of many transcripts in the genome (1), including its own transcript (2). However, premature termination of sense *FLC* transcription has not emerged as the predominant mechanism for repression of *FLC* and proximally polyadenylated *FLC* transcripts were rare in seedlings (1). Instead, FCA was found to promote proximal polyadenylation of *COOLAIR* antisense transcripts at *FLC* (3). This resulted in H3K4 demethylation and H3K27me3 accumulation (4, 5), creating a chromatin environment supporting low transcriptional firing and slow elongation of both *FLC* sense and antisense transcription (6).

The major activator of *FLC* is FRI, with loss-of-function *FRI* alleles accounting for the evolution of many rapid cycling accessions, including Col-0 (7, 8). FRI is part of a COMPASS-like complex that binds near the *FLC* promoter and generates a chromatin environment promoting high transcription and fast elongation (9, 10). FRI acts as a scaffold for LEC2 and FUS3 to enable resetting of *FLC* in the embryo and induce an epigenetically stable high expression state (11).

How the initial antagonistic relationship of FCA and FRI on *FLC* is established has so far been unclear. The expression of endogenous *FCA* is unaffected by *FRI* and vice versa (2). However, the balance of their activities can be modulated through removal of the autofeedback regulation of FCA, which overcomes FRI-induced late flowering (2). Here, we show that they antagonistically regulate polyadenylation site usage of the *FLC* nascent transcript within an early developmental window during embryo development. This establishes an expression state that is then maintained by a Polycomb mechanism during the rest of development.

## Results

### Regulation of *FLC* Polyadenylation Site Usage during Embryogenesis.

**Fig. 1.**
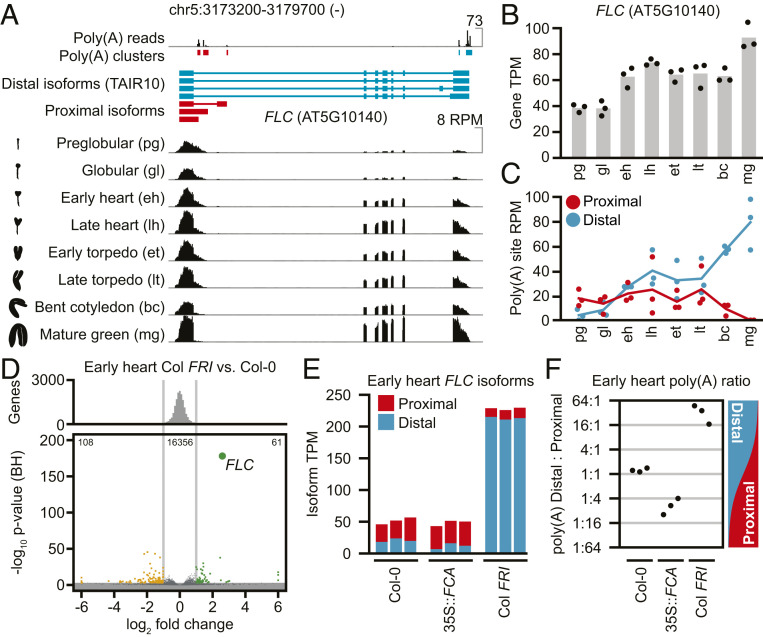
*FLC* polyadenylation site usage changes during embryo development. (*A*, *Top*) Distribution of reads with untemplated 3′-terminal poly(A) sequences across eight timepoints of embryo development and grouped into clusters. (*A*, *Middle*) Transcript models corresponding to full-length (distal) *FLC* and proximally polyadenylated (proximal) *FLC*. (*A*, *Bottom*) Mean RNA-seq read coverage at *FLC* from three biological replicates at eight embryo stages. (*B*) Mean transcripts per million (TPM) of *FLC* across the embryonic time series. (*C*) Poly(A) reads per million (RPM) contained in proximal and distal poly(A) clusters, respectively. (*D*) Differential gene expression between Col *FRI* and Col-0 embryos; genes more than twofold significantly higher (green) or lower (orange) in Col *FRI* embryos are marked (adjusted *P* value <10^−3^, DEseq2). (*E*) Estimated abundance of proximal and distal FLC isoforms in three biological replicates of Col-0, 35S::*FCA*, and Col *FRI* early heart embryos. (*F*) Distal:proximal poly(A) ratio in the same samples as in *E*, calculated as log_2_[proximal poly(A) RPM/distal poly(A) RPM].

We used RNA sequencing (RNA-seq) to analyze *FLC* expression and map sense transcript polyadenylation sites across eight stages of *Arabidopsis* Col-0 embryo development (12). Three proximally polyadenylated *FLC* isoforms were identified that terminated within the first intron of *FLC* near the Polycomb nucleation region (13) (Fig. 1*A* and Dataset S1). Proximal *FLC* isoforms accounted for over 80% of poly(A) reads in preglobular Col-0 embryos (Fig. 1 *B* and *C*). From the early heart stage onwards, a majority of *FLC* transcripts were canonically spliced and used distal polyadenylation sites, and by the mature green stage, poly(A) reads from proximal *FLC* were undetectable.

To understand how the repressor FCA and activator FRIGIDA modulate *FLC* we analyzed polyadenylation site usage in dissected early heart stage embryos from Col-0 (mutant for *FRIGIDA*) (7), Col *FRI*, and transgenic Col-0 carrying a *35S::FCA* overexpression construct (4). The latter was chosen over an *fca* mutant because of functional redundancy between *fca* and *fpa* (1). *FLC* was the most significantly up-regulated gene in Col *FRI* embryos, reaching higher transcript abundance at the early heart stage than any timepoint measured during Col-0 embryo development (Fig. 1 *D* and *E* and Dataset S2) (11). This higher *FLC* expression was associated with distal polyadenylation site usage; while total transcript abundance was 4.4-fold higher, distal isoforms were 10-fold higher and proximal isoforms were half as abundant as in Col-0 (Fig. 1*E*). Proximal poly(A) reads roughly equalled distal reads in Col-0, but distal poly(A) usage outweighed proximal poly(A) reads 20 to 1 in Col *FRI* embryos (Fig. 1*F* and Dataset S2). No RNA-seq reads could be unambiguously attributed to *COOLAIR* antisense transcripts.

There was a shift from distal to proximal polyadenylation in the *FLC* transcript in *35S::FCA* embryos compared to Col-0 (Fig. 1 *E* and *F*), further reducing functional *FLC* expression. FCA thus represses *FLC* expression in Col-0 by promoting proximal polyadenylation via a mechanism enhanced by *35S::FCA*. The shift to predominantly distal polyadenylation site usage at *FLC* by the end of embryogenesis in Col-0 correlates with reduction of functional FCA through increased FCA autofeedback negative regulation (Dataset S3) (1). FRI thus antagonizes this FCA-induced proximal polyadenylation by promoting use of the distal *FLC* polyadenylation site early in embryogenesis.

### FRI Is Required in Early Embryogenesis to Fully Delay Flowering.

**Fig. 2.**
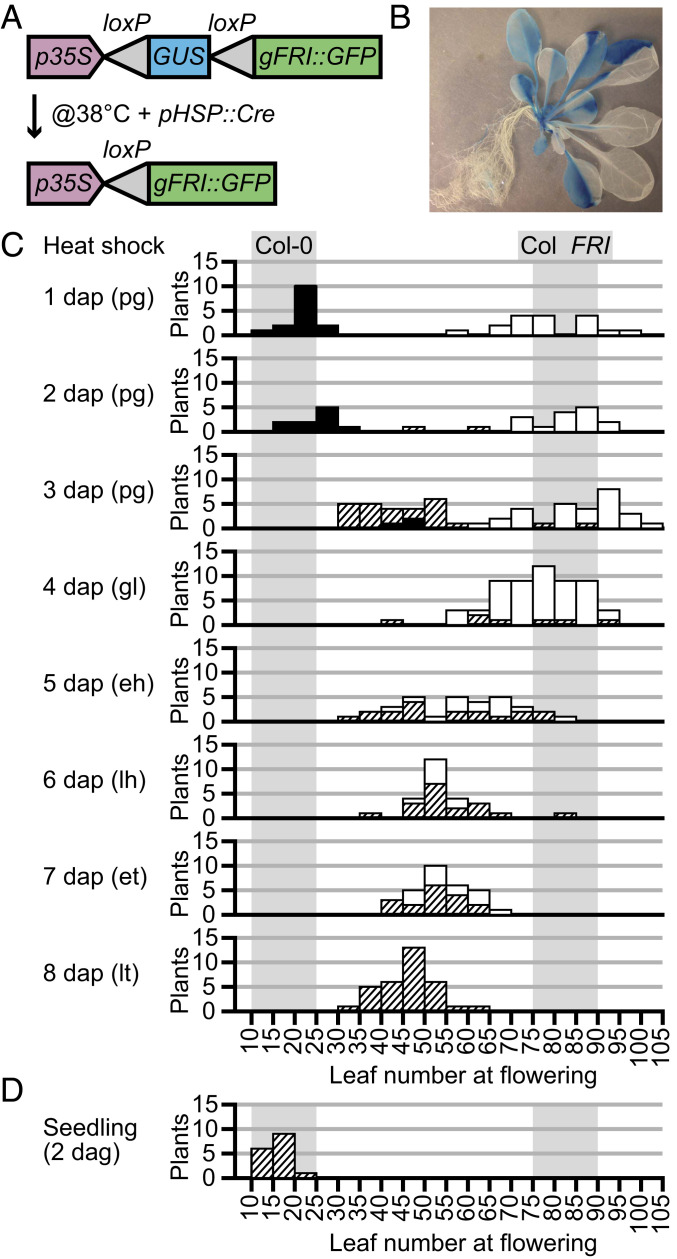
Induction of *FRIGIDA* sectors during embryogenesis and influence on flowering time. (*A*) Schematic of the 35S:lox-GUS-lox-FRI-GFP construct before and after heatshock. (*B*) GUS staining in a mosaic plant heat shocked as a 2-d-old seedling. (*C*) Flowering time assessed by final leaf number in plants that were heat shocked at different stages of embryo development. White bars, plants with FRI-expressing sectors covering all leaves; hatched bars, plants with FRI-expressing sectors and FRI-lacking sectors; black bars, plants with no FRI-expressing sectors. Shaded regions show the range of control plants: Columbia-0 (*fri*) or plants transformed with an active *FRI* allele. (*D*) Leaf number at flowering of *35S:lox-GUS-lox-FRI-GFP* plants heat shocked as 2-d-old seedlings for 30 min. Control ranges are shaded as in *C*.

To further address the timing of the antagonism between FCA and FRI, we established a sector analysis system to address when FRI function is required to fully delay flowering. To generate FRI positive sectors we introduced a *35S:lox-GUS-lox-FRI-GFP* construct containing a *GUS* reporter fusion flanked by two loxP sites between the 35S promoter and FRI-GFP into a heatshock-inducible *Cre-loxP* recombinase line (Fig. 2*A*). The Cre recombinase catalyses recombination between the two *loxP* sites leading to the excision of the GUS reporter enabling FRI to be expressed under the control of the 35S CaMV promoter. FRIGIDA active sectors are thus negative for GUS (Fig. 2*B*).

Heatshock treatment was applied to a developmental series of embryos ranging from 1 to 8 d after pollination (dap). Seeds from heatshocked embryos were sown and the flowering time was analyzed based on total leaf number (Fig. 2*C*). When plants began to flower, they were harvested and stained to detect the presence of the GUS reporter, thereby allowing the size and location of FRI-expressing/GUS-lacking sectors to be determined. FRI-expressing sectors generated in preglobular and globular embryos resulted in individuals that flowered as late as the Col *FRI* control (Fig. 2). By contrast, when *FRI* was induced in older embryos the resulting plants did not flower as late. Comparison of plants that had similar sized FRI-expressing sectors but generated at different times supported the conclusion that FRI induction before the heart stages of embryo development causes maximum delay in flowering. Use of the very strong estrogen-inducible promoter may account for delayed flowering seen in a previous study when FRI is induced in 5-d-old seedlings (14). In our experiments, heatshock treatments of seedlings 2 d after germination generated FRI-expressing sectors, but again, this had no effect on flowering time.

## Discussion

Mechanistic dissection of *FLC* regulation has previously focused on postembryonic development. *FLC* expression is low in Col-0 seedlings through an FCA-induced antisense-mediated chromatin silencing mechanism. Here, we now find that FCA promotes proximal polyadenylation of the sense nascent *FLC* transcript in Col-0 early embryos. This is developmentally specific as by the end of embryogenesis and during the rest of postembryonic development, the sense transcript is polyadenylated predominantly at the distal site.

We found that FRIGIDA up-regulates *FLC* expression by changing *FLC* polyadenylation site usage in heart stage embryos. Overexpression of FCA promotes proximal polyadenylation of the sense transcript, whereas FRI shifts polyadenylation to the distal site. Based on our sector analysis data, FRI antagonizes FCA-induced proximal polyadenylation during early embryogenesis. How FRI blocks the premature polyadenylation is an interesting question. FRI promotes a high transcriptional state and a chromatin environment involving H3K36me3 and H3K4me3, which promotes transcriptional initiation and faster elongation (10). The faster elongation may influence polyadenylation site choice through kinetic coupling of transcription and cotranscriptional processing, with stronger 3′-polyadenylation sites being chosen if already transcribed (15). Alternatively, FRI could act as an antiterminator more directly by regulating what associates with the RNA Pol II carboxy terminal domain via a transcriptional checkpoint.

By integrating these data with our previous mechanistic understanding, we propose that a low transcriptional state is induced by FCA-mediated proximal polyadenylation of *FLC* to establish a specific *FLC* chromatin state during early embryo development. This would be propagated in seedlings by the antisense-mediated Polycomb inheritance mechanism (5). A separation of an establishment phase and a maintenance phase fits with the original paradigm established for Polycomb-silenced genes. However, if an active FRIGIDA is present, *FLC* proximal polyadenylation is prevented in the embryo, the switch to the Polycomb-repressed state does not occur, and the locus is maintained in a high transcriptional state that persists through vegetative development (10). Opposing functions of cotranscriptional regulators at a very specific developmental stage thus set the quantitative expression state of *FLC*.

## Materials and Methods

All methods are given in *SI Appendix*. Materials and associated protocols are available from corresponding authors.

## Supplementary Material

Supplementary File

Supplementary File

Supplementary File

Supplementary File

Supplementary File

## Data Availability

RNA-seq data have been deposited in Gene Expression Omnibus (GEO) (GSE166728).
