# Supplementary material for: Antagonistic activities of cotranscriptional regulators within an early developmental window set FLC expression level
Source: Proc Natl Acad Sci U S A. 2021 Apr 20;118(17):e2102753118. doi: 10.1073/pnas.2102753118 (PMC8092400; doi:10.1073/pnas.2102753118)
Supplement: Supplementary File [file pnas.2102753118.sapp.pdf]

Supporting Methods

Plant material and growth conditions

Seeds were surface sterilized and stratified at 4° C for three days before being sown on soil. Col *FRI* seeds (1) were initially grown on ½× MS media without sucrose for 7 days in long day-condition (21° C, 16h light/ 8h dark cycle) and transferred to 4° C under short-day conditions (8h light/16h dark cycle) for 6 weeks of vernalization prior to transferring the seedlings to the soil. All plants were grown in a climate-controlled growth chamber at 20° C to 22° C under a 16h light/8h dark cycle.

RNA extraction, cDNA library preparation, and next-generation sequencing

RNA extraction from dissected embryos, as well as Smart-seq2 (SSII) cDNA and NGS library preparation were as previously described (2) with the following modifications. Twenty heart stage embryos were dissected from Col-0, Col *FRI* and *35S::FCA* genotypes, and NGS libraries were prepared using non-commercial Tn5 for library tagmentation. NGS libraries were sequenced on a NextSeq550 in paired end-75 base mode. All sequencing data generated in this study have been submitted to the National Center for Biotechnology Information Gene Expression Omnibus (NCBI GEO, https://www.ncbi.nlm.nih.gov/geo/) under accession number GSE166728.

RNA-seq analysis of *FLC* polyadenylation sites

Positions of mRNA cleavage and polyadenylation (poly(A) sites) were inferred genome-wide by mapping Smart-seq2 reads. Strings of 9 or more 3′-terminal A's were removed from reads which were labeled as putative poly(A) reads. All reads were aligned to the TAIR10 genome using STAR v2.7.3a (3). Putative poly(A) reads that aligned immediately upstream of a purine-rich sequence (15 nt of ≥80% A or G) were discarded as likely artifacts of oligo-dT mispriming. A set of high-confidence poly(A) clusters was generated by comparing poly(A) read density to non-poly(A) read density using EndGraph, a statistical model previously established for RNA 5′ ends (4). Poly(A) clusters that overlapped in at least two of three biological replicates in at least one stage of the embryo time series were retained and combined with nanoPARE *bed_consensus.py*. Proximal *FLC* isoform models were manually constructed based on aligned read coverage and consensus poly(A) sites. Proximal and distal *FLC* isoform abundances were estimated with Kallisto v0.46.0 (5) using TAIR10 annotations plus proximal *FLC* isoforms, and by counting poly(A) reads that align to proximal and distal polyadenylation sites as reads per million mapped poly(A) reads (RPM). Differential gene expression was performed using DEseq2 (6) on the Kallisto gene counts table (Supplemental Dataset 4).

Heatshock induction of FRI sectors

An Arabidopsis line C17-2-2 (hsp18.2:Cre) containing the heatshock promoter upstream of the Cre-recombinase gene in Columbia was a gift from Prof. Robert Sablowski (John Innes Centre) and has been described (7). Line C2473 contained both 35S:lox-GUS-lox-FRI-GFP and *FLC-luciferase* (8) in Columbia. *35S:lox-GUS-lox-FRI-GFP* was generated by cloning a gFRI-GFP fusion into pRS044 (provided by Robert Sablowski) downstream of the 35S:lox-GUS-lox sequence.

C17-2-2 plants (carrying hsp18.2::Cre) were crossed with line C2473 carrying 35S:lox-GUS-lox-FRI-GFP on eight consecutive days. For heat shock, 24 hours after the eighth pollination, the inflorescences were submerged in a 38ºC water bath for 15 minutes. Immediately after heatshock plants were transferred to standard growth conditions and seeds were collected when ripe. To determine the embryonic stages corresponding to each time point, one set of siliques from each time series was not heatshocked but harvested, fixed in 90% acetone for 1 hour, cleared in chloral hydrate,

and embryos were inspected with Nomarski optics. The seeds resulting from the heatshocked plants were grown and flowering time determined. The pattern of FRI-expressing sectors in each plant was then assessed by GUS staining. White sectors where GUS expression was absent indicated regions where FRI was under the control of the 35S promoter (and *vice versa*).

Supplemental Datasets

Supplemental Dataset 1. Annotation and quantification of proximally polyadenylated *FLC* across eight stages of *Arabidopsis* Col-0 embryo development

Supplemental Dataset 2. RNA-seq quantification of *FLC* isoforms in heart stage embryos from Col-0, Col *FRI*, and 35S::FCA

Supplemental Dataset 3. *FCA* autoregulation changes during embryogenesis

Supplemental Dataset 4. Differentially expressed genes in Col *FRI* vs. Col-0 early heart stage embryos

References:

1) Lee, S. D. Michaels, A. S. Masshardt, R. M. Amasino, The late-flowering phenotype of FRIGIDA and mutations in *LUMINIDEPENDENS* is suppressed in the Landsberg *erecta* strain of Arabidopsis. Plant J. 6, 903–909 (1994). doi:10.1046/j.1365-313X.1994.6060903.x
2) F. Hofmann, M.A. Schon, M.D. Nodine, The embryonic transcriptome of *Arabidopsis thaliana*. *Plant Reprod*. 32: 77-91 (2019).
3) A. Dobin, C. A. Davis, F. Schlesinger, J. Drenkow, C. Zalenski, S. Jha, P. Batut, M. Chaisson, T. R. Gingeras, STAR: ultrafast universal RNA-seq aligner. *Bioinformatics* 29, 15-21 (2013).
4) M. A. Schon, M. J. Kellner, A. Plotnikova, F. Hofmann, M. D. Nodine, NanoPARE: parallel analysis of RNA 5' ends from low-input RNA. *Genome Res.* 28, 1931-1942 (2018)
5) N. L. Bray, H. Pimentel, P. Melsted, L. Pachter, Near-optimal probabilistic RNA-seq quantification. *Nat. Biotechnol.* 34, 525-527 (2016)
6) M. I. Love, W. Huber, S. Anders, Moderated estimation of fold change and dispersion for RNA-seq data with DESeq2. *Genome Biol.* 15, 550 (2014)
7) J.L. Gallois, C. Woodward, R. Sablowski, Combined SHOOT MERISTEMLESS and WUSCHEL trigger ectopic organogenesis in Arabidopsis. *Development* 129, 3201-3217 (2002).
8) F. Liu, V. Quesada, P. Crevillen, I. Baürle, S. Swiezewski, C. Dean, The Arabidopsis RNA binding protein FCA requires a Lysine Specific Demethylase 1 homologue to down-regulate *FLC*. *Molecular Cell* 28, 398-407 (2007).
